# A cross-sectional study on dietary assessment, oral hygiene behavior, and oral health status of adolescent girls

**DOI:** 10.3389/fnut.2022.973241

**Published:** 2022-10-05

**Authors:** Yuni Mahriani, Ratna Indriyanti, Iwan Ahmad Musnamirwan, Arlette Suzy Setiawan

**Affiliations:** ^1^Resident of Pediatric Dentistry, Faculty of Dentistry, Universitas Padjadjaran, Bandung, Indonesia; ^2^Department of Paediatric Dentistry, Faculty of Dentistry, Universitas Padjadjaran, Bandung, Indonesia

**Keywords:** adolescent school girls, oral health status, oral health behavior, diet assessment, stunting and skinniness

## Abstract

Adolescents are a population group that is vulnerable to nutritional problems other than toddlers, especially young women. Special attention to the dietary issues of adolescent girls needs to be obtained along with the increase in the adolescent population in Indonesia because this affects the growth and development of the body and will impact adult nutrition problems. The purpose of the study was to analyze the relationship between diet assessment and oral health status of adolescent girls, the relationship between oral hygiene behavior and oral health status of adolescent girls, and the simultaneous relationship between dietary assessment and oral hygiene behavior with the oral health status of adolescent girls. Analytical research using the survey method was conducted on 96 young women in two junior high schools in Bandung. Assessment of diet seen from eating behavior and anthropometric examination. Eating behavior using the Adolescent Food Habit Checklist Index questionnaire and anthropometric investigations were carried out by looking at body height, body weight, and Mid Upper Arm Circumference using the standards from the Indonesian Minister of Health Regulation 2020. Oral hygiene behavior using the Oral Hygiene Behavior Index questionnaire. Oral health status using the Dental Health Status Assessment. The results were statistically analyzed with Spearman’s Rank Correlation, and Multiple Linear Regression tests showed no significant relationship between dietary assessment and oral health status (eating behavior with a *p*-value = 0.429 and anthropometric examination with a *p*-value = 0.262). A significant association between oral hygiene behavior and oral health status, with a *p*-value of 0.003, while there is no simultaneous relationship between diet assessment and oral hygiene behavior with oral health status, with multiple *r*^2^ = 13.2%.

## Introduction

In Indonesia, the prevalence of small infants from the small mother group (body height =150 cm) is currently 34.8% (1). The study of Demographic Health Surveys in 54 countries found that for every 1 cm less in mother’s height, the risk of underweight and stunting in children under five increased. In an analysis of 52 of 54 countries (96%), the association between maternal height and stunting was statistically significant (2). However, the association between maternal and infant length may reflect both genetic background and the environmental determinants and developmental experiences mothers experience during childhood, maturation, and subsequent offspring growth. For example, women with short stature have a higher risk of having babies with lower birth weight (LBW), and teenage pregnancy may also increase the risk of LBW events. In addition, stunting seems to be interpreted as malnutrition themselves, but especially in Indonesia, it was shown that stunting is more influenced by education and social structure than nutrition (3–6). There is a new concept of the modern view of growth regulation that involves social-economic-political-emotional (SEPE), which was explained by Bogin (7). This modern view will be explained why stunting prevention needs to involved not only health parameters.

Prevention of stunting has become a national priority so that the younger generation in Indonesia can grow and develop in the best possible way. The government has issued the National Strategy to Accelerate Stunting Prevention guide for central and local governments to implement stunting prevention efforts. The National Stunting Strategy includes specific and sensitive nutritional interventions to improve nutrition. Specific nutrition interventions aim to address nutrition-related issues directly through the health sector. At the same time, sensitive nutritional interventions aim to address non-health issues contributing to stunting, such as providing clean water, food security, and health insurance (8).

A group at high risk for nutritional problems is adolescents (9). With the increasing adolescent population in Indonesia, adolescent nutrition must be given special attention as it affects physical growth and development and will impact adult nutrition. Nutritional problems in adolescents, whether under- or over-nutrition, can adversely affect public health. Adolescent malnutrition will have a negative impact on public health, such as reduced physical fitness, reduced productivity, and even the fertility of adolescents themselves, especially young women (10).

One of the methods used to assess a person’s nutritional status is dietary assessment. Direct assessment of diet is by looking at eating behavior and anthropometry. Adolescent eating behavior has received increasing attention in recent years amid claims that many adolescents have a poor diet, including high levels of fatty foods and low intakes of fruit and vegetables. Variations in adolescent dietary intake are likely to reflect available diets, values and circumstances of parents, school and peers, and their motivation. If adolescents’ eating habits/behavior are poor, it will affect the clinical condition that indicates a nutritional deficiency, thus affecting their anthropometry (11).

Oral health plays a fundamental role in overall health and wellbeing. Oral health has a significant impact, significantly on the quality of life (12). Many factors affect a person’s oral health: socioeconomic status, education, living and school environment, and individual behavior. Eating behavior and oral health have a synergistic and dynamic relationship (13). Diet, nutritional composition, and poor oral and dental conditions will interact and have a role in the formation play a role in caries’ formation motion of sweet foods contained in individual snack patterns are the main factors of a cariogenic diet. Oral health is closely related to oral hygiene because oral hygiene is a primary factor in creating oral health. Oral hygiene can determine a person’s level of oral health. Young women need to consider maintaining nutritional status and oral hygiene, starting from pre-wedding preparations, especially brides-to-be, to preconception, gestation, and postpartum, which may directly impact the fetus later on; what does it contain (14). Therefore, this study aimed to analyze the association between nutritional assessment, oral hygiene behaviors, and oral health status in adolescent girls. It is hypothesized that there is a simultaneous relationship between the evaluation of diet and oral hygiene behavior with the oral health status of adolescent girls.

## Materials and methods

### Study design and participant

Analytical observational school-based studies with a cross-sectional design were used, and survey data were collected through questionnaires and anthropometric surveys. Two schools were selected due to being located at a stunting locus [based on the Decree of the Mayor of Bandung regarding stunting loci in Bandung City (15)]. Participants were recruited by simple random sampling. The subjects of this study were middle school girls who met the following criteria: (i) were in the age range of 10–15 years, (ii) had no chronic systemic disease, and (iii) were willing to participate in the study (proofed by parental signed informed consent).

### Sample size

In this study, correlation analysis was used. The formula used to determine the sample size was the formula used to determine the sample size using correlation analysis, (i) type I error (Zα) = set at 5% with a one-way hypothesis, so Zα = 1.96; (ii) type II error (Zβ) = set at 10% with a one-way hypothesis, then Zβ = 0.84; and (iii) research correlation coefficient (r) = 0.3. Based on the calculation of the sample size formula above, a minimum of 84 people is required (16).


$$n={\left[\frac{\left(Z\,\alpha +Z\,\beta \right)}{0,5\text{ln[}\frac{1+r}{1-\text{r}}]}\right]}^{2}+3$$

### Dietary assessment

Dietary assessments will be assessed using adolescent girls’ dietary habits and anthropometric surveys. Eating behavior is a student’s repetitive pattern of eating habits, which can be evaluated using a questionnaire. At the same time, anthropometry is assessed by examining the student’s height, including height, weight, and mean upper arm circumference (MUAC). The dietary assessment tool consisted of the Adolescent Eating Habits Checklist (AFHC) questionnaire (7), adapted into Indonesian for this study and tested on 64 adolescents, which is not included in this study. The reliability of the tested questionnaire leads to a Cronbach’s alpha = 0.86.

The AFHC questionnaire contains 23 “correct” or “incorrect” or “not applicable to me” questions. Responses indicating healthy behaviors are scored as 1. Final scores were adjusted for “not applicable” and missing responses using the following formula: AFHC score = number of “healthy” responses × (18/number of items answered). The measurement scale uses the Guttman scale, and the last level multiplied by 100 becomes the interval scale. The examples of the questions for instance, if I am having lunch away from home, I often choose a low-fat option (11).

Anthropometric examination by calculating the formula body-mass-index (BMI) per age and MUAC. BMI category (kg/m2): thin underweight, weight level < 17.00; Mild weight loss 17.00–18.49; Normal 18.50–24.99; Overweight/ Mildly overweight 25.00–26.99; Excess weight level > 27.00, then the BMI results are entered in the Z-Score formula, while the MUAC threshold used is 23.5 cm. Anthropometric examination measuring scale using a ratio scale. The procedure measuring MUAC was to determine the mid-point between the elbow and the shoulder (acromion and olecranon). Placing the colored tape measure around the left arm (the arm should be relaxed and hang down the side of the body). Measure the MUAC while ensuring that the tape neither pinches the arm nor is left loose. Read the measurement from the window of the tape or from the tape. Record the MUAC to the nearest 0.1 cm or 1 mm. If a measurement in the green zone means the child is properly nourished; a measurement in the yellow zone means that the child is at risk of malnutrition; a measurement in the red zone means that the child is acutely malnourished (17).

### Oral hygiene behavior

Oral hygiene behaviors were participants’ verbal care behaviors assessed using a questionnaire. The Oral Hygiene Behavior Questionnaire was developed based on the theory of planned behavior (18) and tested on 50 adolescents that are not included in the main study (Cronbach’s alpha = 0.842). The questionnaire contains 17 items assessing attitudes, subjective norms, and perceived behavioral control. The response score is 0–5. Total scores range from 0 to 85, with higher scores indicating positive oral hygiene behaviors. The measurement scale adopts the Likert scale, and the final result is multiplied by 100 to become the interval scale.

### Oral health status

Oral health status is an oral health condition felt by the participant assessed from the questionnaire. Because it was still a pandemic, oral health status was measured using a self-reported dental health status assessment questionnaire (19) trans-adapted into Indonesian (Cronbach’s alpha = 0.758). The original questionnaire contained 10 statement items to assess caries risk, but only seven items remained after undergoing trans-adaptation and pre-test. Responses were scored from 0 to 1. The total score ranged from 0 to 7, with higher scores indicating individuals with better health status. The measurement scale uses a Likert scale. The final score times 100 becomes an interval scale.

### Data collection

Data collection took place in April 2022. Prior to the day of data collection, informed consent was given to the parents of the students who explained the course of the research. In addition, parents are asked to fill in the child’s health data, whether the child has a particular disease or disability. It is necessary to determine the confounding factors in this study. Adolescents with a history of chronic systemic disease were excluded from participation. Aside from the child’s health history, the parents also asked questions regarding social-economic status.

Participants previously received instructions on how to complete the questionnaire. After participants understood, they simultaneously met all questionnaires (AFHC, Oral Hygiene Behavior Questionnaire, Oral Health Status Questionnaire) in two separate rooms. A research assistant accompanied each. After completing the questionnaires, the researchers (principal investigators) administered anthropometric measurements to the participants, whom research assistants assisted. Anthropometric measurements include weighing with a digital scale to measure body weight. All participant were weight with their school uniform clothes on and barefooted. Body height was measured using microtoise stature meter with an accuracy of 0.1 cm (20).

### Data analysis

The collected data were processed descriptively and analytically. For descriptive data, statistical measures of counts and percentages for categorical and numerical data, by plotting mean, standard deviation, median, and range. When the data are not normally distributed, analyze the data using statistical tests with Pearson’s correlation analysis or Spearman’s rank correlation. Normality tests used Kolmogorov-Smirnov data, while multiple linear regression analysis was used to analyze simultaneous associations between dietary ratings, oral hygiene behaviors, and oral health status. Use a *p*-value < 0.05 to determine the significance of test results.

### Research ethical aspects

This study has been ethically reviewed by the Research Ethics Committee of Padjadjaran University No: 463/UN6.KEP/EC/2022.

## Results

Ninety-six students completed questionnaires and anthropometric measurements. Table 1 shows the characteristics of the participants. As seen from the characteristics table, the average age of adolescent girls in both schools is 13.8 years old, with a range of 12–15 years. Family socioeconomic status was predominantly middle class (47.9%); most educational qualifications were classified as low 43.8% (secondary school and below), and anthropometric findings were described as normal (85.4%) based on BMI/age and based on MUAC was normal (52.1%).

**TABLE 1:** Characteristics of participants (*n* = 96).

Characteristics	Statistics value
**1. Age (year)**	
• Mean (SD)	13.8 (0.78)
• Median	14
• Range	12–15
**2. Parental social-economic status**	
• Low	42 (43.8%)
• Middle	46 (47.9%)
• High	8 (8.3%)
**3. Parental education**	
• Low	41 (42.7%)
• Middle	35 (36.5%)
• Tinggi	20 (20.8%)
**4. BMI/Age**	
• Thinness	10 (10.4%)
• Normal	82 (85.4%)
• Overweight	4 (4.2%)
**5. Mid Upper Arm Circumference**	
• Thinness	39 (40.6%)
• Normal	50 (52.1%)
• Overweight	7 (7.3%)

A description of the variables studied; the oral hygiene behavior score, the eating behavior score, and the oral health status score are presented in Table 2. As seen from the table, the data for the three variables based on the normality test yielded a *p*-value < 0.05; the data are not normally distributed, leading to non-parametric statistics being used for further analysis.

**TABLE 2:** Statistical description of nutritional (diet) behavior and oral hygiene behavior scores by adolescent oral health status.

Variable	Mean	SD	Median	Range	Normality test data (*p*-value*)
1. Oral hygiene behavior	75.2	7.20	75	58–85	0.041
2. Diet behavior	9.69	4.00	10	2–22	0.017
3. Oral health status	3.11	1.44	3	0–6	<0.001

*Based on the Kolmogorov-Smirnov test. SD, standard deviation. Significant if *p*-value < 0.05.

The statistical analysis by Spearman rank correlation in Table 3 shows that the correlation between oral health status score and oral hygiene behavior score was *r* = 0.300; *p* = 0.003, which means that the higher the oral hygiene behavior score, consequently increase the oral health condition score. While the relationship between eating behavior and oral health status in adolescent girls showed a non-significant correlation (*r* = 0.082; *p* = 0.429).

**TABLE 3:** Correlation of nutritional assessments (eating behaviors) and oral hygiene behaviors with oral health status in adolescents.

Correlation of oral health status scores with	Correlation coefficient (r_s_)	*P*-value
Oral hygiene behavior score	0.300	0.003
Eating behavior score	0.082	0.429

r_s_, Spearman rank correlation. Significant if *p*-value < 0.05.

Table 4 presents the comparative analysis of oral health status scores of various characteristics of adolescent girls. From the table, it shows that there is a difference in oral health status scores; the socioeconomic status of the family (*p* = 0.033), the most significant oral health status score, in the middle socioeconomic status with a median = 4, while the lower and upper socioeconomic status with a median = 3. For parental education, there seems to be a tendency that the higher the parental education, the higher the oral health status, although statistically not significant (*p* = 0.088/*p* > 0.05). Comparison of oral health status scores based on dietary assessment from anthropometric examinations (BMI/Age and MUAC) obtained a *p*-value = 0.262, which showed no significant difference or in other words anthropometric examination is not significantly related to the oral health status of adolescent girls.

**TABLE 4:** Comparison of oral health status scores based on characteristics.

Characteristics	Oral health score			*P*-value*
		
	Subjects (n)	Median	Range	
**1. Parental social-economic**				
• Low	42	3	1–6	0.033
• Middle	46	4	0–6	
• High	8	3	1–5	
**2. Parental education:**				
• Low	41	3	0–6	0.088
• Middle	35	3	0–6	
• High	20	3.5	1–6	
**3. Dietary assessment BMI/ age**				
• Thinness	10	2	1–5	0.262
• Normal	82	3	0–6	
• Overweight	4	3.5	1–5	
**4. Dietary assessment MUAC**				
• Thinness	39	3	1–6	0.262
• Normal	50	3	0–6	
• Overweight	7	3	3–6	

*Based on Kruskal-Wallis’s test.

Furthermore, to analyze the relationship between dietary assessment and oral hygiene behavior from various variables (because the data is not homogeneous) with oral health status scores using multiple linear regression analysis. The variables involved in this analysis have a *p*-value < 0.25 from the results of the bivariable study; parents’ education, socioeconomic, eating behavior scores, and oral hygiene scores. The results of multiple linear regression analysis are presented in Table 5.

**TABLE 5:** Relationship of oral health status scores with various variables based on multiple linear regression.

Variable	B-Coefficient	SE (B)	t	*P*-value
**Initial models**				
Parent’s education	0.315	0.217	1.449	0.151
Socio-economic status	0.292	0.267	1.093	0.277
Eating behavior score	0.022	0.038	0.585	0.560
Oral hygiene behavior score	0.058	0.020	2.820	0.006
**Final models**				
Parent’s education	0.436	0.181	2.411	0.018
Oral hygiene behavior score	0.060	0.019	3.105	0.003
Constanta	–2.181			

For the final model multiple *r* = 0.363 (*r*^2^ = 13.2%). B-Coefficient, regression coefficient; SE(B), Standard of Error in coefficient B; *t*, B-coefficient /SE(B).

Table 5 presents the analysis of the relationship between oral health status scores based on multiple linear regression of various variables. The first step is to estimate the model or any variables studied because the variables studied are dietary assessments (eating behavior and anthropometric examination) with oral health status with data that varies on research subjects or is not homogeneous, so that confounding variables such as status socioeconomic and education are also included in the initial model in this multiple linear regression analysis. The second step is to carry out classical assumptions, namely to obtain a model estimated to be feasible or not in interpreting the influence of the independent variable on the dependent variable. The variables involved in the final model have a *p*-value < 0.25. From the four initial models that have been analyzed, the final model that relates to oral health status scores is parental education and oral hygiene behavior scores with a positive B coefficient, meaning that the more parents’ education and oral hygiene scores increase the oral health status score. The magnitude of the coefficient of determination (r2 multiple) = 13.2%; it means that 13.2% of the variation of the oral health status score is influenced by parental education and oral hygiene behavior scores, and the remaining 86.8% is influenced by other factors not studied. This analysis concludes that the hypothesis is rejected, which states that there is a simultaneous correlation between the evaluation of diet and oral hygiene behavior with the oral health status of adolescent girls.

## Discussion

The characteristics of the research subjects obtained based on the inclusion and exclusion criteria were 96 people with an average age of 13.8 years with a range of 12–15 years. According to WHO (21), the age of 10–15 is the early adolescent stage. At this stage, it can be used as a basis for nutritional counseling and planning for youth education programs (21). Some things need to be considered in young women, which is maintaining nutritional status and oral hygiene, starting with preparing themselves from pre-wedding, especially the bride-to-be, until the pre-pregnancy, pregnancy, and postnatal period (22). Based on the 2018 Indonesian Research Data report, 57.6% of Indonesians experience dental and oral health problems in the form of dental caries and periodontal disease. A significant increase in dental and oral issues occurs in adolescents aged 12–18 (23). In addition, the Ministry of Health also released the Effective Medical Demand (EMD) value in adolescent girls, which was higher (9.1) than in teenage boys (7.1) (24). Therefore, adolescent girls are more prone to dental and oral problems than boys.

The results of the correlation analysis of eating behavior scores in this study showed a non-significant relationship; This means that good eating behavior does not necessarily indicate good health status. Assessment of diet (eating behavior) is related to a person’s nutritional status. The better a person’s nutritional status, the better the dental health status. This is in line with Budisuari’s study which states that a person’s eating habits or patterns can affect the occurrence of caries, especially if a person tends to consume sugar which is the leading cause of caries. The level of sugar consumption has increased overall in developing countries, and the increase in the prevalence of dental caries in developing countries has been ascribed to the rise in sugar consumption (25). Adolescents begin to decide what to eat without relying on their parents anymore. They also tend to be craving between meals and are very interested in consuming sugary snacks, such as chocolate, candy, and carbonated drinks, which triggers an increase in caries (26). The existence of sweet foods, snacks, and carbonated beverages inside and outside the school complex has reached students to buy and consume them, the interest of teenagers also supports this at this age who are more interested in the taste and appearance of food than its nutritional value (27), but in this study, the eating behavior of adolescent girls was not significantly associated with oral health status. This is understandable because eating behavior is not the only one of the factors that can affect oral health status. Oral health status is influenced by the interaction of four factors: behavior, environment, health services, and genetics. In developing countries such as Indonesia, behavior is indeed the most dominant factor in influencing the status of dental and oral health (28).

In this study, anthropometric examination was not significantly associated with oral health status in adolescent girls. The correlation of anthropometric examination and oral health can be explained as follows; the anthropometric examination is related to a person’s nutritional status, and this is in line with the study by Busman and Atigah which states that nutritional status is a sign of the body’s appearance caused by a balance between nutrient intake and health expenditure as seen through the variables of height, weight, and growth. Lack of nutrition is caused by various factors, including infectious diseases and food intake (29). Another study from Ratnasari and Junaidi (30), the effect of dental caries can cause disturbances in the digestive process and eating difficulties that generate growth and development disorders, and vice versa, meaning that the better a person’s nutritional status, the better the dental health status. Nutrition is essential in developing and defending oral health, especially teeth and gingiva. Healthy or unhealthy conditions of teeth and gingiva can affect food intake. In adolescents with dental caries, there is often a disturbance in the information about food substances which is a factor causing lack of nutrition, and it can cause a decrease in the body’s biological function or malnutrition (29). The oral health is part of the health of the body that affects each other. In this study, anthropometric assessments of adolescent girls were not significantly associated with oral health status. This is consistent with Nurlaila and Herwati a survey conducted in Karangantu District, Banten Province, that found no significant association between nutritional status and dental caries in school children aged 9–14. This situation is more because cariogenic food can have a direct impact as the cause of dental caries if it is supported by a state of low tooth resistance, a form of saliva that is less than normal and dense (31).

Adolescents are not only physically mature but also cognitively and emotionally. They seek identity, strive to be independent and acceptable, and pay a great interest to their self-appearance. These changes significantly impact eating behavior and skipping between meals, snacking, and café’s dine in. These habits are further influenced by families, groups, and the media. In addition, the influence of the physical environment, such as air that has experienced air pollution and extreme temperatures, as well as physical activity, although generally considered positive, can give stress to the body, affecting a person’s nutritional needs. In addition, the influence of the non-physical environment, such as the family and community environment, socio-cultural factors, and socioeconomic factors, also affect the nutritional status of adolescents (26).

The results of the correlation analysis of oral health status scores with oral hygiene behavior scores showed that there was a significant relationship (rs = 0.300; *p* = 0.003). This means that the higher the oral hygiene score, the higher the oral health status score. Good oral hygiene behavior will affect oral health status. Study by Budisuari stated that tooth brushing behavior affects the occurrence of caries. This is related to the process of caries itself, where if sucrose stays in the mouth for a long time and is not cleaned immediately, it will lead to the possibility of caries. This follows Gustafson’s opinion, stating that sugar consumption increases caries attack activity. The most significant risk is if sugar is eaten in a form that is easily attached and not cleaned immediately (32). Another study that supports this opinion conducted by Stephen, stated that after consuming carbohydrates, the pH of the mouth will drop in 5–10 min to 5.5, which is acidic, but will return to normal within 30–45 min, with the addition of sucrose in the form of drinks, bread, chocolate, caramel and candy between meals can cause an increase in caries activity. Still, if consumed at mealtimes, the formation rate will be reduced, or in other word, no caries will form (33). Two approaches can be taken to improve one’s oral hygiene. First, the individual approach through accelerating the increase in the ability to help oneself behave in a healthy life. For individuals who suffer from systemic disorders and periodontal disease, the behavior of maintaining oral hygiene should be intensified. The individual approach includes age, knowledge, children’s dependents, type of work, expenses, sources of costs, distance to the dentist, smoking habits, attitudes, and actions. Meanwhile, another study stated that individual factors include oral hygiene, frequency of brushing teeth, and eating acidic foods (PH < 7) (34).

The second approach, the contextual approach, includes the ratio of dentists to the population, the ratio of hospitals to residents, the ratio of dental clinics to residents, and the ratio of health centers to residents. Other contextual factors are the source of drinking water and the acidity of the water. Piped drinking water in all areas and distribution is under the supervision of the local PDAM, as well as water acidity tests by the health center are carried out periodically. In addition, other contextual factors also include the availability of dental nurses, dentists, per capita health budget, and other environmental factors that significantly influence the prevalence of oral dental disease (34). From the results of multiple linear regression analysis, the final model that relates to the oral health status score is the oral hygiene behavior score, with a positive B coefficient, meaning that the higher the oral hygiene score, the higher the oral health status score. From the magnitude of the coefficient of determination (r2 multiple) = 13.2%, it means that 13.2% variation of the score of oral health status is influenced by parental education and oral hygiene behavior scores, and the remaining 86.8% is influenced by other factors not studied. H L Blum stated that the degree of health of a person or society is influenced by four factors, namely: environment, behavior, heredity, and health services. According to Laurence Green, three factors influence a person’s behavior, namely predisposing factors, supporting factors, and reinforcing factors. Health behavior is divided into knowledge, attitude, and action. Knowledge is a very important domain for the formation of one’s actions. Factors that affect a person’s knowledge are education, occupation, age, interests, experience, and ease of getting information (35).

Adolescents’ dental and oral health maintenance is often neglected, while adolescents are also vulnerable to oral health problems during puberty. In addition, many bad habits of teenagers can cause damage to the teeth and mouth. These bad habits include laziness of night time tooth-brushing, the practice of consuming sweet foods, and the pattern of drinking sweet or soda beverage (36). Contextual factors that influence oral health status are environmental factors and followed by behavioral factors. Environmental factors affect the oral health status of about 40%, and behavioral factors affect about 30%, so it can be said that environmental and behavioral factors will affect more than two-thirds of oral health status in the community (34). Personal factors that play a role in improving oral health status include age and gender. In addition, education, occupation, economic status, knowledge, attitudes, and actions also play a role in improving oral health status. Patterns of healthy living behavior of individuals and families, non-smoking behavior followed by the ability to help oneself also affect oral health. Research in the UK states that social factors, on individual factors, are the main determinants of dental and oral health status. Individual factors were obtained from the frequency of tooth brushing, oral hygiene, and eating habits of vinegar/acidic food (PH < 7) (5, 34).

Nutrition and oral health have a two-way relationship; proper nutrition is essential in maintaining oral health. Otherwise, oral health is also crucial for maintaining adequate nutritional intake. Untreated dental caries can cause pain, causing not only eating problems but also speech and sleep problems. Furthermore, these eating disorders can have long-term impacts such as iron deficiency, anemia, and malnutrition (37–39). In a study by Morenike et al. (40) in Nigeria, it was stated that malnutrition could lead to enamel hypoplasia, which creates a niche environment for plaque retention. Furthermore, malnutrition results in hypofunction of the salivary glands, changing saliva composition and reducing its buffering capacity, thereby increasing caries formation. In addition, the study found that nutritional deficiencies were associated with oral hygiene. Previous studies also conducted in Nigeria have identified that oral hygiene is associated with a higher prevalence of caries. In contrast, other studies have highlighted the increased prevalence of caries due to poor oral hygiene (40). Although nutrition and oral hygiene can affect the oral health status of young women, which will have an impact on their quality of life (one of which is malnutrition which later as a mother-to-be can cause the risk of stunting in her child), many other factors that have been described previously, can also affect oral health status in adolescent girls. Therefore, the limitations of this study are the existence of variables or other factors related to oral health, both individually and family dimensions, as well as overall, as well as the use of self-assessment that is not supported by clinical examinations due to the COVID-19 pandemic.

## Conclusion

There is a relationship between oral hygiene behavior and adolescent girls’ oral health status. However, the simultaneous relationship between dietary assessment and oral hygiene behavior with the oral health status of adolescent girls was not proven—similarly, the relationship between dietary assessment and oral health status of adolescent girls.

The above indicates the need for further research on other factors that can be related to oral health so that it can complement the research that has been done so that it can become a study in stunting prevention, as well as dental and oral examinations on research subjects.

Oral health and nutrition education with health promotion and nutrition counseling. It is hoped that there will be a collaboration with other professions, including doctors, parents, schools, nutritionists, health educators, and the youth health community who can help regulate adolescent nutrition. In addition, it is recommended to visit a dental polyclinic, a dentist’s private practice or a health center that must be done at least every 6 months.

## Data availability statement

The original contributions presented in this study are included in the article/supplementary material, further inquiries can be directed to the corresponding author.

## Ethics statement

The studies involving human participants were reviewed and approved by the Research Ethics Committee of Padjadjaran University No: 463/UN6.KEP/EC/2022. Written informed consent to participate in this study was provided by the participants’ legal guardian/next of kin.

## Author contributions

AS and RI conceptualized the study design. YM and AS collected the data. YM analyzed the data. IM, AS, and YM conceptualized the first draft. RI reviewed the first draft. AS finalized the writing. All authors contributed to the article and approved the submitted version.
